# Evaluating a community academic partnership to advance equity-focused cancer genetic implementation research: a qualitative analysis of partner perspectives

**DOI:** 10.3389/fpubh.2025.1569414

**Published:** 2025-05-30

**Authors:** Ariana Naaseh, Meera Muthukrishnan, Isabel Temosihue, Erin L. Linnenbringer

**Affiliations:** Division of Public Health Sciences, Department of Surgery, Washington University School of Medicine, St. Louis, MO, United States

**Keywords:** community engagement, CBPR, community participation, partnership, public health, cancer genetics, health equity

## Abstract

**Background:**

Forming community academic partnerships (CAPs) can increase the applicability, translation, and dissemination of implementation research focused on addressing health inequities within the community setting. We aimed to explore community and academic partners’ perspectives on their participation in a novel, multi-disciplinary cancer genetic equity CAP focused on developing a multi-level intervention for breast, ovarian, prostate, and pancreas cancers.

**Methods:**

We conducted semi-structured interviews with CAP members. Questions addressed partners’ motivations for participation in the CAP, feedback about the partnership, and opportunities for future CAPs. All interviews were audio-recorded, de-identified, transcribed, and then coded inductively by 2 analysts to identify relevant themes.

**Results:**

8 CAP members participated. We identified four main themes including motivations to participate and continue engagement in the CAP, perceived CAP successes, perceived CAP challenges, and suggestions and opportunities for improvement. Participants described a variety of motivations, including learning more about cancer genetics and helping patients and communities. Participants valued the multi-disciplinary collaboration and having facilitated partnership discussions. Challenges included ambiguity of expectations for roles and differing perspectives between providers and community members, sometimes leading to frustrations in discussing solutions to potential barriers. Participants described several suggestions for improving future partnerships, such as more clearly defining expectations for participant roles, being able to create a strong vision and targeted approach, bringing their counterparts into clinical and community spaces to better share differing perspectives, and involving leadership stakeholders in the partnership to help address healthcare system barriers.

**Discussion:**

Overall, community and academic members were motivated to participate and engage in a CAP to improve cancer genetic equity.

## Introduction

Traditionally, research partnerships between communities and academic institutions have been unilateral in nature and have not routinely incorporated community stakeholder input (1). The lack of community consideration when developing research areas of focus and study design can prove to be incredibly problematic when translating academic pursuits to impactful community change. In recent years, community- based participatory research (CBPR) and community-academic partnerships (CAP) have been developed to increase collaboration and ensure that the communities’ voices are reflected in both the identification of public and population health-based concerns and study design to address them (2, 3). Ensuring that marginalized communities are included in conversations addressing health inequities is a central tenant of CAPs (4). CBPR is utilized to ensure better quality research and increase the applicability, translation, and dissemination of results within the community setting (5). This does not come without challenges, but has been proven feasible (6–8).

Although utilizing CAPs to conduct CBPR has garnered significant attention, it was not until 2015 that quantitative methods were created to assess the effects of engagement within the participating population (9, 10). Prior qualitative research has demonstrated aspects of a successful partnership included developing faculty-community relationships, articulating common goals tailored around local priorities, and a recognition of community value (11). However, there are minimal published reports both detailing how best practices have been applied in the establishment of CAPs to guide intervention selection, adaptation, and implementation research and how to evaluate these partnerships (12). We aimed to fill this gap in partnership evaluation by exploring our community and academic partners’ perspectives on their motivations to participate and sustain engagement in a novel, multi-disciplinary cancer genetic equity CAP focused on developing a multi-level intervention for breast, ovarian, prostate, and pancreas cancer.

## Materials and methods

### Study setting

This study was approved by the Washington University in St. Louis of Medicine (WUSM) Institutional Review Board. We utilized a qualitative research design and conducted semi-structured interviews with participants of our cancer genetic equity focused CAP. Partnership members convened for 8, 2-hr meetings over a period of 13 months (January 2023 – February 2024) to review current gaps in local cancer genetic services for African Americans, select a target for intervention, and develop an intervention prototype based on existing framework and evidence-based interventions. This work was guided by Nápoles and Stewart’s transcreation framework for community-engaged interventions to reduce health disparities, along with established community based participatory research principles (13, 14).

### Participant recruitment

The CAP consisted of 11 members, 6 community members who included both cancer survivors and mutation carriers along with 5 clinical and academic members representing specialties including Gastroenterology, Obstetrics and Gynecology, Oncology, and Genetic Counseling. Community members were recruited via the alumni network of the Community Research Fellows Training program and outreach to individuals who previously expressed interest in related cancer research (15). Siteman Cancer Center-affiliated clinicians with experience in germline cancer genetic testing were also invited to participate. CAP recruitment was closed when a balanced number of community and academic partners agreed to attend the first meeting.

All 9 partners who remained engaged throughout the entirety of the 13-month partnership were invited to participate in our study via email invitation. 8 agreed to participate in semi-structured interviews.

### Data collection

Participants were individually interviewed for 30–60 min each via Zoom (Zoom Video Communications Inc., 2016) in April 2024. Interviews were audio-recorded, de-identified, and professionally transcribed. Interviews were conducted by a research team member who was familiar with the partnership design and structure, but was not actively involved in its implementation, to ensure participants felt comfortable sharing openly in a safe and confidential environment. All community participants received a gift card for their participation. Providers were unable to receive compensation due to institutional policies.

### Interview guide

The interview guide was developed by leaders of the partnership based on points raised in the literature regarding CAPs and community based participatory research (CBPR) (5). The interview guide was refined by the research team based on trial interviews prior to using it in actual data collection. Trial interviews with the final interview guide on average occurred for 30-min in duration, therefore participants were recruited for planned 30-min interviews. The interview guide was designed to learn more about the partners motivations for participating in and remaining engaged in the CAP, to obtain feedback about their partnership experience, and to understand potential opportunities for improvement for future CAPs designed by our group or other groups.

### Data analysis and interpretation

All interview transcripts were uploaded into NVivo 14 (QSR International; Doncaster, Australia) for qualitative analysis. Field notes were taken throughout the interview and used to supplement transcription content. Two research team members (AN and MM) conducted a thematic analysis, using both deductive and inductive methods (16). An initial codebook was developed using deductive codes based on the interview questions. Each transcript was individually coded by two study team members (AN and MM) and results compared to ensure reliability. Coding discrepancies were resolved with discussion among the coders, with a third researcher available for discussion if needed. Team members met regularly to discuss emergent codes and further develop the codebook. The code book was iteratively refined using an inductive approach throughout analysis, where inductive codes were derived from the transcripts. Coders individually created summary statements and compiled the summaries into themes. Themes and selected exemplar quotes were reviewed by the study team. Themes were revised until consensus was reached.

## Results

### Participants

Our interview sample included 4 African American community members, 2 of which (50%) were male and 2 (50%) were female. The sample included 2 White academic clinical members and 2 Asian academic clinical members, of which 3 (75%) were female and 1 (25%) was male. Quotes are attributed to participants by number and letter code, with P indicating providers and C indicating community members.

Four main themes emerged: motivations to participate and continue engagement in the CAP, perceived CAP successes, perceived CAP challenges, and suggestions and opportunities for improvement (Table 1).

**Table 1:** Themes and representative quotes.

Description of theme	Exemplar quotes
Theme 1: motivations to participate and continue engagement	
Passion for cancer genetics and bringing awareness of the benefits of genetic testing and improving healthcare and outcomes	“I think just the ability to share what I am seeing on the front lines if you will, or, you know, part of my research had been on developing tools to help facilitate cascade genetic testing and get these tools into the community—and so I was hopeful to share some of that with the group and see if we could move that forward.” – Academic Provider
Personal history with cancer	“I am also a two-time breast cancer survivor, and I have the gene mutation, so genetics and hereditary cancer is dear to my heart.” – Community Member
Helping others and their communities	“People were very serious about it. The people that were in charge of those meetings—I know how much time they dedicated them selves, you know, in helping other people, you know. And, I was really, really, really proud of that, you know. To see how much people care about others when they could have very easily—you know…I say, ‘Wow. That’s really dedication.’” – Community Member
Creating lasting and meaningful change	“There was definitely a sense of, ‘Gosh, this is a big issue and, you know, this requires a lot.’ And while you know, multiples make up the whole, right, that—you know, every—it’s important for multiple different organizations and approaches and attempts to kind of change the system, right? That it did seem like a lot with that one particular effort to try to make big lasting meaningful change like we all wanted to see.” – Academic Provider
Theme 2: perceived partnership successes	
Diversity in background and experiences of the participants	“I think the nice thing about this group of providers that was engaged too, was that it kind of crossed a lot of different departments and divisions and different disease specialties, and I really value getting to work with my colleagues across departments at WashU. That also really appealed to me that we were going to be spending more time together and pushing our passion for genetics forward, and we each come in with our various sets of barriers from different divisions and different departments.” – Academic Provider
Diversity in background and experiences of the participants	“There were multiple different stakeholders that were going to be participating, so whether it’s community members, other physicians in sort of different disciplines than mine to kind of bounce ideas off of. I think it’s also been a really rich experience, just to have the facilitated discussions between <facilitator> and-and everyone to just think about all the different angles that I might not be aware of from my perspective.” – Academic Provider
Safe space for discussion	“I definitely think it was a safe space….I think some of us got emotional because of the health disparities and things of that nature, but it was a safe space” – Community Member
Theme 3: perceived partnership challenges	
Wanted more than the clinical perspective	“We do not just want clinical expertise. You are experts of your own community and your lives and when you are coming in and showing up throughout the entire process, you bring other perspectives too.” – Academic Provider
Providers were not as engaged	“I saw all the community members engaged. I was engaged in most of the meetings, but then the provider side of things was a little lacking. I’m sorry. I’m saying this as you are recording, but really, the provider side was lacking, and I understand. You are providers, you have meetings, you have urgent things come up, but when everyone else is showing up to every other meeting, it feels a little like the issue is being trivialized. Like, yes, this is on my agenda, but it’s an afterthought.” – Community Member
Tension between the providers and community members	“There was just an interesting - somewhat of a we did not quite merge in the middle, but I think it was interesting to feel that sort of tension between community and provider in terms of like what was possible, and what should be possible, and ‘Why cannot this be possible?’ and ‘aren’t you a doctor, and do not you care?’ type of thing. And that wasn’t real, I did not feel that necessarily, but I feel that deeply.” – Academic Provider
Theme 4: suggestions and opportunities for improvement	
Include decision makers in partnership	“I think that’s something really interesting to have the people who would be the decision-makers be able to be the ones in the room who are number one, just hearing what the community members are saying—which, like you said, is—it’s really compelling, it’s hard to like be like, “Yeah, no that’s not right,” or, “That’s not fair,” because you agree, you are like, “These are all reasonable things that people should have access to and should be given,” but having that come from potentially someone else might help and have the providers not wear so many different hats.” – Academic Provider
Bring community members to clinic to provide a new perspective	“It might be reasonable to bring the community into the clinic in a way that they could see it that way too because like we would be in a conference room that is more of an inspiring space I think than—you know, it’s a neutral ground I guess, but I do not know, I just wonder about having one meeting over here at the hospital or the clinic or even one in a community space to all of these spots where care is provided could be helpful.” – Academic Provider

### Motivations to participate and continue engagement

Participants described a variety of things that motivated them to participate. Both providers and community members wanted to learn more about cancer genetics to better help their patient and communities, especially underserved patients/community members.

*“I think that’s what inspires a lot of us—just seeing disparities like this in my own clinical practice. Knowing that I’m frequently maybe not serving my under-resourced and underserved patients as well and really knowing how prevalent prostate cancer is in our Black patients, our African American patients, and wanting to be part of the change as it were.” – 1P.*

Participants mentioned a passion for cancer genetics and bringing awareness of the benefits of genetic testing and improving healthcare and outcomes.

*It was the genetics’ piece that caught my eye because there are a lot of rare cancers that if someone got genetic counseling, they—not that they would not get it, but they would be forewarned. Right? Before they get in the thick of it, or before it’s like stage IV or stage V, before they ever figure somethin’ out. So I always like to learn, and I liked to learn different perspectives, and so I just figure if I joined a board maybe it could help me understand what I can do better as an advocate.” -6C.*

Some participants wanted to share their personal or family cancer experiences. Most participants wanted to collaborate and build partnerships to help their communities.

*“Because I think without community partners we really—we cannot just put that research out into the academic world and expect it to be implemented easily without partnering with our communities.” - 2P.*

*“I do believe if we come together we can make it happen as far as genetic counselors being able to be visible in a community. I know it takes funding to do all of these things so I do believe in what we are trying to do and accomplish.” – 4C.*

Once participation began, members of the partnership saw many future directions for a positive impact from partnership activities. At times the scope of the partnership’s goals felt broad which made defining a clear way to have an impact challenging. Partners also noted that while making impactful change is challenging, frustrating, and a long process, this partnership helped bring transparency to that.

*“Yeah, and it’s just kind of a reminder that all of this takes so long, and to change anything that needs to be changed is such a long process.”-2P.*

Other motivations include financial compensation for participation and academic opportunities. Providers were excited by the prospect of publishing peer-reviewed manuscripts and having the potential to change clinical practice. Overall, the passion and dedication the partnership members put forth for the cause helped motivate and excite engagement throughout the process.

### Perceived partnership successes

Many participants had positive feedback about the partnership. They enjoyed the experience overall and some felt it was well structured. The facilitator, who is an experienced strategic change professional with an extensive community network, had a positive impact and helped create a safe space.

*“Most facilitators might just be doing stuff, but she is awesome, because she has the wisdom to really piece things together. So that even helped to really drive down deeper level of understanding for those who are not self-motivated to get there.” -7C.*

Participants felt that the partnership research team was invested in the success and mission of the partnership. The partnership was a good balance between encouraging and having defined goals. Some felt the partnership was a great opportunity to learn from both types of participants, but also about cancer genetics and systemic barriers to providing healthcare. Also, there was great diversity in the background and experience of the participants – as patients, community members, and organization leaders.

*“I think overall it was a really good* var*ying group because some of us run organizations. There were some patients there, and there was some people that had organizations but really were trying to get more knowledge around genetics.”-6C.*

Participants valued the different perspectives that were part of the collaboration (patient, provider, community) as well as being able to collaborate with different departments/divisions at our institution. There were some conflicting perspectives between providers and community members. While only participant mentioned being overwhelmed at the beginning, overall participants felt that people were nice and understanding and that they had a safe space to share their thoughts. They also felt their viewpoints were respected.

*“Well, at the beginning, I felt overwhelmed by all that knowledge around me. And, I was able to communicate with a couple of people in that class that were very nice to me and very understanding.”-8C.*

### Perceived partnership challenges

A number of challenges arose for partners in the scope of the work of the partnership and the structure of the partnership. Participants acknowledged the partnership was developed to address a very important and heavy issue. Questions of addressing racial equity arose and challenged participants about a much larger and deeply entrenched issue within the healthcare system that the partnership was working against. Though participants felt that the scope of what the partnership was trying to accomplish was very challenging, they still felt their partnership experience was rewarding.

*“Just realizing how entrenched inequity and inequality are in the healthcare system and the medical system and this in particular, and I feel like in some ways this is really just emblematic of so much other disparity, right? And just the sense of kind of frustration about how poorly we are serving a large segment of our population, you know? And so just running up against that whenever we’d talk about these things we are just thinking about how to attempt to mitigate this problem that really just is one sign of a larger problem”-1P.*

In terms of the structure of the partnership, some partners found it challenging to ensure everyone’s voices were heard and to move partnership agenda items forward. They also found it challenging to get the whole partnership on the same page about what was accomplished at the last meeting given the infrequency of the meetings. When recaps were provided, they felt that took away from time spent on the current meeting agenda.

*“I think the gaps between the meetings were too long for me to go from one meeting to another. I felt like the second meeting, we spent almost 45 min recapping what happened in the previous meeting. If we had more frequent meetings, that would not have been necessary.”-3P.*

Logistical challenges included scheduling. This was difficult for community partners especially as it occurred during peak traffic hours.

*“The travel sometimes was challenging because it’s the time people get off of work and just the traveling was a little problematic. I would just say the traffic sometimes got to me.”-4C.*

Additionally, it was challenging for providers not to be compensated for the time and effort they put into the partnership, when that expectation was set at the start of the partnership.

*“Financial compensation was one of the reasons why I participated, and when that was also taken away, it was a little demotivating to be like, “Hey. Why? Why am I being faulted for being part of the institution that I’m trying to help?”-3P.*

Participants expressed that there were challenges with how roles were defined in that they were either not defined enough or that perhaps the perceived role of each partner as “community” or “partner” led to unnecessary siloing of opinions and perspectives.

*“I think the-the clear demarcation of, yes, academic versus community partners may have caused that or contributed to a divide, perceived divide, possible divide. I do not know.”-3P.*

Some community participants felt that they did not belong as much because they had no clinical expertise, but those who felt validated in their roles as survivors and connectors to the community found feelings of belonging.

*“Many times, like I said, I felt like I did not belong because I did not have the medical background like a few of those people did, you know, but at least I was an example of survival.”-8C.*

Some partners expressed that at times they felt their content expertise and experience was not relevant – that at times the topic became too specific, and it was difficult to keep things broad and applicable to all.

*“The reason that I stopped engaging in the last several meetings was, like, the project was focusing very far away from GI and GI genetics or whatever. So, I was like, I do not really feel my personal expertise or experience here is all that relevant to where this is going.”-5P.*

There was also tension between the providers and community members in that providers felt that intimate knowledge of the barriers faced by providers in the healthcare system was lacking by community members and contributed to frustrations in discussions about health care delivery and service models. It was also felt that while the partnership structure was such that all members felt comfortable imagining future directions and solutions, that there was delayed and minimal discussion on previous interventions and barriers to provide context to the partnership’s goals.

*“I guess one thing in reflecting about this is that the community partners or the people from the community just did not seem to quite understand the barrier. We listened to a lot of things that I think we already have been sort of depressed about, or not necessarily like, “Yeah, we tried that,” “Yeah, we tried that,” “Yeah, we have tried that,” but I do not know, obviously we need to rethink and re-envision how to do it. But I think a lot of us—obviously, I can only speak for myself—but I sort of sensed from different providers too this sort of like, “Yeah, that would be great,” but we are sort of stuck in a system that has not allowed us to move things forward.”-2P.*

*“One of the things I also realized was that a lot of our community partners did not realize how much economics actually plays into the decision making. And that was like, I felt bad on some levels to break it to them that a lot of these decisions are not being driven by equity necessarily in their heart but by economics.”-5P.*

*“I think that some of the people in there were thinking and hoping too high. You know? They do not understand how the people work in the real world. You know? It’s going to be themself first. You know? You know, they are in the business of making money. Everything else is just secondary. So.” -7C.*

*“Because most of the time, when you listen to [the providers] during the meeting, it’s as if there is a roadblock and there’s nothing anybody can do about it. And I do not see it like that. I think that these doctors can actually do something in removing these barriers that patients often experience, which is why minorities do not even care about coming out to participate in the first place.”* – *7C.*

### Suggestions and opportunities for improvement

Participants shared feedback and several suggestions for improving future partnerships. Several of these addressed potential solutions for previously noted challenges, such as more clearly defining expectations for participant roles and sharing member expertise and backgrounds ahead of time.

*“Maybe in retrospect it would have been helpful to have defined at the outset. To sort of say, you know, ‘This is the—,’ obviously not to pigeonhole folks, right, but to say in the beginning, ‘This is the expected role of-of these particular individuals and this is how we hope or plan for you in particular to contribute so that we can make sure that each perspective is well represented.’”-1P.*

Having patient advocates for each main disease group would also be helpful in creating solutions that are appropriate for all. Many participants also advocated for better focus by creating a strong vision and targeted approach.

*“We have to have a strong vision, targeted approach, focus and move towards it involving the communities and the stakeholders. That is the way anything involving structural movement succeeds. If you do not have your vision strong and you do not have a narrow focus for your outcome, you may just try, but even if you think you succeed from an awareness point of view, we sometimes really want real results.”-7C.*

To address impact challenges, participants suggested bringing the community members into the clinics while also getting clinical members into the communities.

*“Just come in the community so you can see what I’m saying.”-4C.*

Participants expressed that it was challenging to develop a concrete plan to move forward in improving cancer genetic equity without important stakeholders in the room. To address this, participants suggested involving hospital leadership in the partnership to discuss what interventions could be practically implemented, based on available resources.

*“There’s only so much that you, me, <Name> and <Name> can do, right, within our specific silos, and we are limited by the resources that we have. So, there’s the elephant in the room is <Business> leadership, and whether or not they are actually interested in committing resources to address this. So, in the future what I recommend is to have their involvement, at that level. You know, have <Name> come and join one of these things.”-5P.*

Solutions for logistical challenges were conflicting. Some advocated for more frequent, shorter meetings, to keep momentum going, while others preferred less frequent and longer meetings. A few participants suggested having breakout meetings for different deliverables with smaller groups.

*“Once you get to a certain stage have little breakouts based on these different objectives and have more meetings, maybe more in tune with a smaller group of people who are focused on specific outshoot of this.”-5P.*

Other practical solutions included sending email recaps for each meeting and sending references to the group before meetings, so meeting time is not used to review it. Several participants also suggested having a Zoom option for meetings, to encourage participation if not able to attend in person.

## Discussion

Interviewing partnership members following the completion of the planned CAP meetings enabled understanding of community member and provider needs and perspectives that will lead to deeper connections and support the development and implementation of future interventions and projects. All members of the partnership acknowledged the value of the mission of the partnership and appreciated the intentionality with which it was structured to optimize its potential community impact. Both the partners and the research team exited their partnership experience with new ideas and possibilities for future collaboration.

Some of the challenges the team encountered within the partnership have been previously described in the literature. While most partners felt comfortable in the CAP environment due to prior preparation, not all participants were familiar with CAP or CBPR prior to engaging with partnership activities. Structured training prior to beginning partnership activities has been demonstrated to be feasible and effective (5, 17). Throughout the partnership, there were challenges with communication and understanding between community and academic partners which have been previously described in the literature. It has been suggested that perhaps this could be mitigated by ensuring transparency at the start of the partnership in each partners roles, skill set, and expertise. Formally documenting such points along with clear expectations can be helpful for partnership success (18). Successful CAPs also introduce the members to best practices to inform the interventions or projects that the CAP undertakes (19). The partnership research team strove to provide this information by conducting a scoping review of cancer genetic focused health equity community interventions in parallel with the CAP meeting schedule. While the CAP was provided an interim summary of the scoping review findings, presenting the final synthesized analysis may have been more useful to our partners and enhanced their participation.

There are also well documented challenges in the literature that were not experienced by the partnership members, including: inconsistent partner participation or membership, mistrust among partners, lack of common language or shared terms among partners, a high burden of activities or tasks, and excessive funding pressures or control struggles (12). Our partnership planning process pre-emptively addressed these issues in several ways. First, the partnership was structured such that the first meetings were focused around creating a shared understanding and common language surrounding health equity and systemic racism within our local community. The facilitator also worked with the CAP to establish partnership norms and values during these meetings and revisited them periodically throughout the subsequent sessions. Furthermore, we heard from our partners throughout the interview process that the presence of a facilitator ensured that trust and respect was maintained and fostered throughout the duration of the partnership. While some partnerships may involve some work outside of the scheduled partnership, ours required minimal pre- and post-work outside of the agreed upon times. Lastly, our funding mechanism did not provide undue pressure toward the goals or outcomes of the partnership.

An important aim of this qualitative exploration was to learn from our partners’ experiences and create even more successful partnerships in the future. Our discussions with our partners revealed several important insights that are supported by prior research. First, successful partnerships incorporate diverse viewpoints and, when focused on implementation, involve key stakeholders. Selection of motivated and engaged partnership members is paramount to the success of a partnership and every effort should be made by the research team to ensure that participants are empowered to represent themselves, their viewpoints, and their own expertise (18). Second, the importance of trust and respect among partners cannot be understated (12). Clear definition of individuals’ roles could help or hinder partnership process, but at minimum, identifying a facilitator who will create a safe and inviting space for the partnership activities is necessary and appreciated by participants, especially those participating from the community (20, 21). Third, well-structured meetings have been identified as a key facilitating factor of CAP success (12). Establishing CAP meeting logistics that worked well for all members was a challenge for our partnership, and our interviews did not clearly identify a best practice framework for defining the structure and the frequency of meetings needed to maintain a productive and efficient partnership experience. Finally, it is important to ensure that those who are providing their expertise within a partnership are compensated for their participation. While we were able to provide our community-based CAP participants with a $150 consultant’s honorarium per 2-hr meeting, our institution did not allow employees to be compensated in the same manner, and our pilot funding was unable to cover salary support for our participating clinicians. While they may benefit from co-authorship on future publications, securing appropriate financial compensation is important for future work.

It has been demonstrated that partners rank the fit of their collaboration with the philosophy of the organization or partnership they are joining as important for their participation in current and future collaborations (22). Our partnership mission was intentionally broad in its scope, incorporating a wide range of community and clinical perspectives on multiple types of potentially hereditary cancers. While every CAP participant shared a goal of increasing equity in cancer genetic services, individual members were particularly invested in the type of cancer that they most identified with, personally or professionally. Additionally, our CAP was not only charged with identifying key opportunities to improve cancer genetic equity in our local community, but to also develop an evidence-based intervention prototype to achieve the CAP’s prioritized outcomes. In some interviews, the challenging and broad nature of our scope was acknowledged as a facilitator for engagement in the partnership experience. However, future partnerships may be even more successful with a more narrow scope or clinical focus.

Our work demonstrates the value of incorporating CAPs in health equity-focused work due to their emphasis on collaboration and diversity of perspectives. With a group of motivated and engaged participants who have clear expectations and appropriate preparation, a CAP can be highly effective. Ongoing challenges with our CAP and others in the literature include defining an actionable scope along with identifying and managing the possible tensions in the relationship between community and academic partners. This qualitative analysis can provide helpful insights and lessons learned to researchers who are designing and establishing a CAP. Our group hopes to incorporate our partners’ feedback in future CAP efforts focused on advancing cancer genetic equity.

## Data Availability

The anonymized data supporting the conclusions of this article will be made available by the authors, with appropriate Institutional Review Board approval.
